# Interferon-targeted therapy with anifrolumab in monogenic pediatric systemic lupus erythematosus due to C1q deficiency: a case report

**DOI:** 10.3389/fped.2026.1877919

**Published:** 2026-07-30

**Authors:** Seham M. Alqahtani, Abdalrahman Mohammed Asiri, Mohammed A. Aljuaid

**Affiliations:** 1 Department of Pediatrics, College of Medicine, Najran University, Najran, Saudi Arabia; 2 Department of Pediatrics, Division of Pediatric Rheumatology, Prince Sultan Medical Military City, Riyadh, Saudi Arabia; 3 Health Research Center, Najran University, Najran, Saudi Arabia

**Keywords:** anifrolumab, C1q deficiency, monogenic pediatric SLE, pediatric rheumatology, systemic lupus erythematosus

## Abstract

Monogenic Pediatric systemic lupus erythematosus (SLE) secondary to complement deficiencies, including C1Q deficiency caused by C1QA mutations, is a rare and severe type of SLE that can be characterized by early onset and refractory disease. Inhibiting the interferon pathway has proved to be an effective treatment option, although there is little evidence in monogenic pediatric SLE. We describe a 10-year-old female with genetically-verified C1Q deficiency who had persistent and severe mucocutaneous disease, with recurrent skin rash, ulcerations, and oral sores, despite long-term immunosuppressive and biologic treatment. After a disease flare, the patient was started on anifrolumab, a monoclonal antibody against type I interferon receptor. She showed significant clinical improvement and full recovery of mucocutaneous lesions, restoration of laboratory parameters and successful discontinuation of corticosteroids after the seventh monthly infusion. It was well tolerated with no serious side effects. The case demonstrates the possible effectiveness and safety of anifrolumab in the treatment of refractory cutaneous manifestations in children with monogenic pediatric SLE, which is linked to interferon pathway dysregulation. Further studies are needed to establish its role in this population.

## Introduction

Monogenic pediatric systemic lupus erythematosus (SLE) is a rare and severe form of childhood-onset SLE that is caused by single-gene defects, most commonly a defect in the complement pathway, C1q deficiency, due to C1QA mutations (1, 2). These patients are usually characterized by early-onset and aggressive diseases with mucocutaneous manifestations and are resistant to standard immunosuppressive treatments. Despite the established role of complement deficiency as a cause of SLE -like disease, the role of interferon pathway dysregulation in this subgroup is not fully understood (3). C1q deficiency disrupts the clearance of apoptotic debris and immune complexes, leading to increased autoantigen exposure and immune dysregulation. This process is strongly associated with activation of the type I interferon pathway, which plays a central role in SLE pathogenesis. ³ Patients often present with prominent mucocutaneous manifestations, including discoid rash, oral ulcers, and photosensitivity, and may exhibit atypical serological profiles.

Anifrolumab is a monoclonal antibody against the type I interferon receptor that has shown effectiveness in moderate-to-severe SLE, especially in cutaneous manifestations (4) but its application in monogenic pediatric SLE has not been clearly defined (5, 6). This case is novel in reporting the effective application of anifrolumab in a child with genetically confirmed C1QA-associated SLE and refractory mucocutaneous disease, which suggests a potential application of targeted interferon blockade in this rare and difficult disease.

## Case presentation

A 10-year-old girl with a known diagnosis of monogenic pediatric SLE secondary to C1q deficiency (C1QA homozygous mutation: c.479G>A, p.Gly157Asp) was followed at our center. She was diagnosed at the age of five years, with a positive family history in her younger sibling.

**Figure 1:**
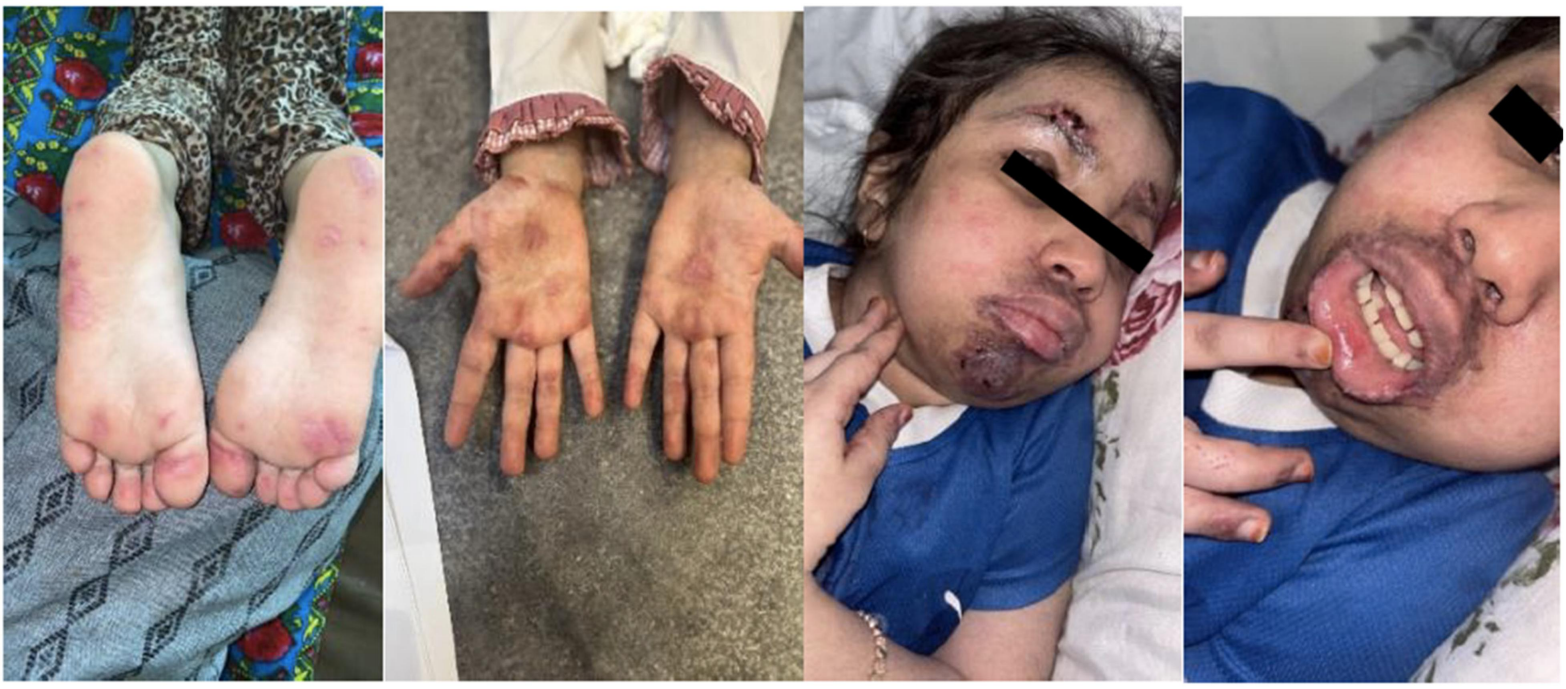
Before starting anifrolumab.

Her treatment history included long-term therapy with low-dose prednisolone, mycophenolate mofetil (600 mg/m^2^ twice daily), and hydroxychloroquine (200 mg daily). She also received belimumab (10 mg/kg every 4 weeks for 10 cycles), followed by tofacitinib (5 mg twice daily) for approximately one year. The patient experienced severe active hypertrophic cutaneous lupus erythematosus six months prior to starting anifrolumab. This condition was characterized by extensive erythematous hyperkeratotic crusted plaques on the scalp, face, ears, perioral region, palms, and soles, along with oral ulceration, alopecia, and areas of post-inflammatory dyspigmentation (Figure 1). Fatigue, arthralgia, and persistent mucocutaneous symptoms were the main features of recurrent flare-ups, which continued to be poorly controlled despite therapy with tofacitinib (5 mg twice daily). To reduce the risk of additive immunosuppression during the transition, tofacitinib was stopped immediately prior to the first anifrolumab infusion due to the insufficient therapeutic response, with no overlap between the two targeted medications. The patient did not present with any neurological symptoms (such as seizures, psychosis, cerebrovascular disease, cranial neuropathy, or peripheral neuropathy), ocular involvement, cardiovascular symptoms (such as myocarditis or pericarditis), pulmonary symptoms (such as pleuritis, pneumonitis, or diffuse alveolar hemorrhage), or gastrointestinal symptoms related to SLE. Renal function was preserved, with a normal serum creatinine and no evidence of active SLE nephritis. The baseline SLEDAI-2K score was 7. Laboratory evaluation revealed leukopenia, positive anti-Smith, anti-SSA, and anti-RNP antibodies, with negative anti-dsDNA and normal complement levels.

**Figure 2:**
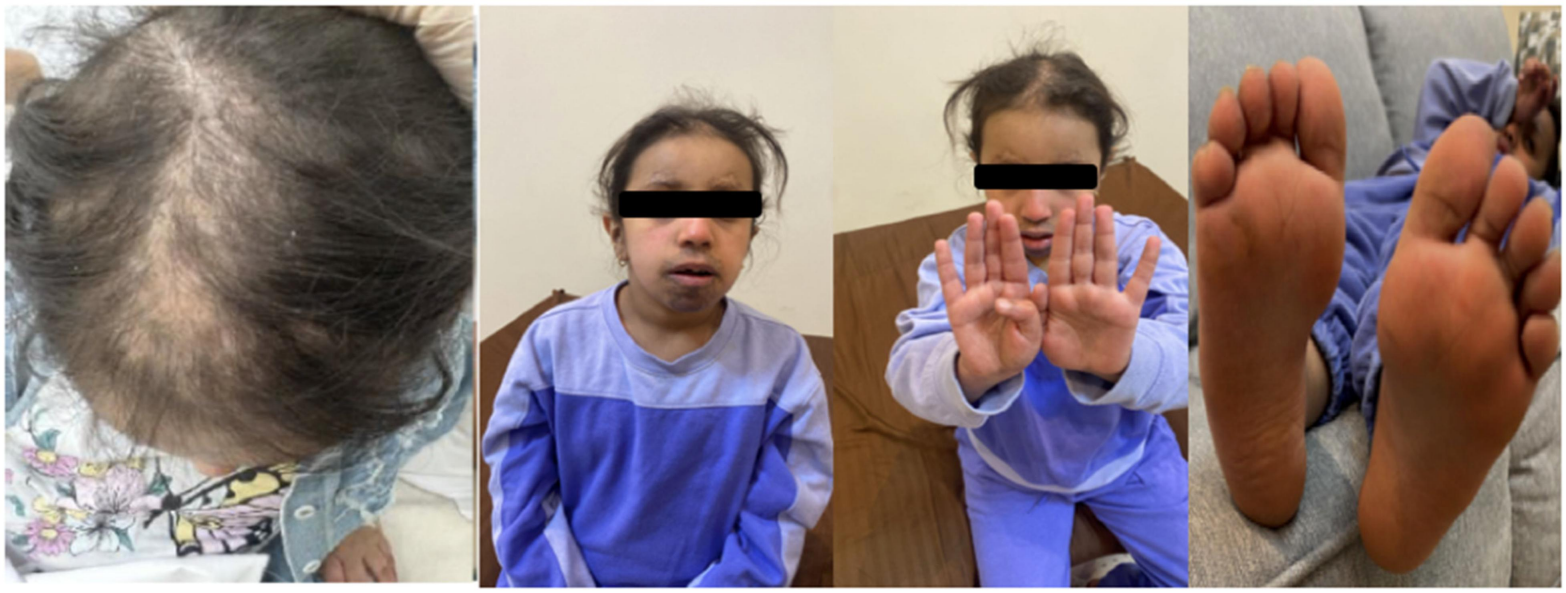
After the seventh dose of anifrolumab.

Given refractory disease, Anifrolumab was administered at a dose of 150 mg intravenously every 4 weeks (approximately 5.5 mg/kg). In the absence of an established pediatric dosing regimen for childhood-onset SLE, this weight-based regimen was selected based on published pediatric experience using monthly anifrolumab at approximately 5.5 mg/kg in patients with type I interferonopathies (7). Before each infusion, the patient received acetaminophen, methylprednisolone, and diphenhydramine as infusion premedication to reduce the risk of infusion-related reactions. No infusion-related reactions were observed throughout treatment. Concomitant prednisolone was continued at 7.5 mg/day at treatment initiation, tapered and successfully discontinued by 3 months while maintaining sustained clinical remission. After the seventh infusion, the patient demonstrated marked clinical improvement, with complete resolution of mucocutaneous manifestations and normalization of hematologic parameters (Figure 2). The patient's longitudinal laboratory findings and disease activity following initiation of anifrolumab are summarized in Table 1.

**Table 1:** Serial laboratory parameters, disease activity, and corticosteroid dose before and after initiation of anifrolumab.

Laboratory parameter	Baseline (0 months)	3 months	6 months
White blood cell count (×10^9^/L)	3	6	6
Hemoglobin (g/dl)	12.08	13.3	12.2
Platelet count (×10^9^/L)	341	458	448
ESR (mm/h)	20	10	8
CRP (mg/L)	0.69	0.75	0.89
C3 (g/L)	1.4	1.36	1.5
C4 (g/L)	0.29	0.27	0.3
CH50 (U/mL)	<10	NR	NR
Anti-dsDNA	negative	negative	negative
SLEDAI-2K	7	0	0
Prednisolone dose (mg/day)	7.5 mg/day	0 mg/day	0 mg/day

WBC, white blood cell count; Hb, hemoglobin; PLT, platelet count; ESR, erythrocyte sedimentation rate; CRP, C-reactive protein; C3, complement component 3; C4, complement component 4; anti-dsDNA, anti-double-stranded DNA antibody; SLEDAI-2K, Systemic Lupus Erythematosus Disease Activity Index 2,000; NR,not repeated;CH50, total classical complement activity.

No disease relapse or significant adverse events were observed during the follow-up period.

**Figure 3:**
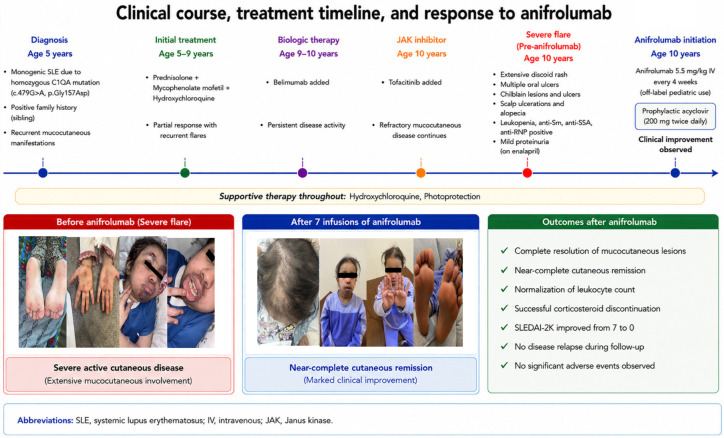
Clinical timeline of disease course and management. The figure illustrates the chronological progression of disease in a pediatric patient with monogenic systemic lupus erythematosus due to C1q deficiency, including initial diagnosis at 5 years of age, recurrent mucocutaneous flares, multiple lines of immunosuppressive and biologic therapies, severe disease flare, and subsequent initiation of anifrolumab (5.5 mg/kg intravenously every 4 weeks, off-label pediatric use). A marked clinical response was observed after treatment initiation, with complete resolution of mucocutaneous manifestations and successful corticosteroid discontinuation after seven infusions.

The clinical course, treatments, and outcomes are summarized in Figure 3, which illustrates the patient's disease trajectory from initial diagnosis to response following anifrolumab therapy.

## Discussion

This is the first pediatric case reported to have shown the clinical effect of anifrolumab in a patient with monogenic pediatric SLE caused by C1q deficiency. In the current case, anifrolumab resulted in significant improvement in refractory mucocutaneous manifestations, which is in line with clinical trials and real-world studies in adults with systemic lupus erythematosus, where significant cutaneous disease activity and glucocorticoid reduction have been reported (5, 8, 9). These results suggest that interferon-targeted therapy can be applied to non-conventional SLE.

As shown in Table 1, clinical improvement after initiation of anifrolumab was accompanied by normalization of disease activity (SLEDAI-2K), a reduction in ESR, stable complement levels, persistently negative anti-dsDNA antibodies, and successful tapering and eventual discontinuation of corticosteroids.

The therapeutic response that has been observed could be attributed to the dysregulation of the type I interferon pathway in the pathogenesis of SLE (8). Even though C1q deficiency is most commonly associated with defective clearance of apoptotic debris and immune complex clearance, it can also play a role in persistent activation of interferon signaling pathways, and thus, promote chronic inflammation (9) Here, the clinical improvement observed, especially in patients with pronounced cutaneous involvement, can be explained by a biologically plausible mechanism of blockade of the type I interferon receptor by anifrolumab.

Moreover, there were no negative consequences. Throughout the course of treatment, anifrolumab was well tolerated; no significant adverse events or infusion-related responses were noted. Before starting treatment, a thorough infectious risk assessment was carried out because type I interferon receptor blockage has been linked to an increased risk of herpes zoster and other viral infections. Pre-treatment screening revealed no signs of active varicella-zoster virus infection, and the patient had received all recommended childhood varicella vaccinations. Prophylactic acyclovir was given during anifrolumab therapy as an extra safety measure. Since there was no clinical suspicion of an active HSV infection, herpes simplex virus serology was not carried out. During follow-up, there were no instances of clinically severe infections or herpesvirus reactivation (6).

The strengths of this case are that the monogenic pediatric SLE is genetically proven, extensive longitudinal follow-up, and the response, both clinical and laboratory, is well documented after the introduction of targeted therapy.

Importantly, this case highlights the potential role of targeted therapies in pediatric patients with refractory SLE, particularly those with monogenic forms of SLE such as C1Q deficiency. A precision medicine approach based on the underlying disease mechanism may improve outcomes in this challenging population, although larger studies are needed to confirm these findings.

Overall, this case supports the use of interferon-targeted therapy as a promising strategy in monogenic pediatric SLE.

However, several limitations should be acknowledged. Although C1Q deficiency is a well-recognized monogenic cause of SLE associated with dysregulated innate immune activation and enhanced type I interferon signaling, biomarkers of interferon pathway activation, including an interferon gene signature and SIGLEC1 expression, were not available and therefore could not be assessed in this patient. Consequently, direct mechanistic confirmation of interferon pathway activation or its inhibition by anifrolumab was not possible. Nevertheless, the observed clinical response is biologically plausible given the established role of type I interferon signaling in monogenic pediatric SLE. In addition, as a single case report, these findings cannot be generalized, and long-term safety and efficacy data for anifrolumab in pediatric monogenic SLE remain limited.

## Conclusion

Anifrolumab may represent an effective and well-tolerated therapeutic option for refractory mucocutaneous manifestations in pediatric patients with monogenic SLE due to C1q deficiency, highlighting the potential role of targeted interferon pathway inhibition in this rare and challenging condition.

## Data Availability

The original contributions presented in this study are included in the article. Further inquiries can be directed to the corresponding author.

## References

[1] Morán Álvarez P Passarelli C Messia V Pardeo M Marasco E Insalaco A. POS0135 Genetical and phenotypical findings of childhood-onset systemic lupus erythematosus. Ann Rheum Dis. (2023) 82:287. 10.1136/annrheumdis-2023-eular.2607

[2] Coss SL Zhou D Chua GT Aziz RA Hoffman RP Wu YL. The complement system and human autoimmune diseases. J Autoimmun. (2023) 137:102979. 10.1016/j.jaut.2022.102979 36535812 PMC10276174

[3] Triaille C Rao NM Rice GI Seabra L Sutherland FJH Bondet V. Hereditary C1Q deficiency is associated with type 1 interferon-pathway activation and a high risk of central nervous system inflammation. J Clin Immunol. (2024) 44:185. 10.1007/s10875-024-01788-5 39196411 PMC11358312

[4] Cingireddy AR Ramini N Cingireddy AR. Evaluation of the efficacy and safety of anifrolumab in moderate-to-severe systemic lupus erythematosus. Cureus. (2024) 16:e63966. 10.7759/cureus.63966 39104974 PMC11299632

[5] Parriel E Bulai-Livideanu C Severino-Freire M Delpuech A Faguer S Belliere J. Rapid clearance of cutaneous lesions with anifrolumab in SLE (systemic lupus erythematosus) and dle (discoid lupus erythematosus). JEADV Clin Pract. (2025) 4:207–15. 10.1002/jvc2.590

[6] Martín-Torregrosa D Mansilla-Polo M Morgado-Carrasco D. [Translated article] use of anifrolumab in systemic lupus erythematosus, cutaneous lupus erythematosus, and other autoimmune dermatoses. Actas Dermosifiliogr. (2025) 116:T55–67. 10.1016/j.ad.2024.10.008 39389344

[7] Kretzschmar G Páez LP Tan Z Wang J Gonzalez L Mugabo CH. Normalized interferon signatures and clinical improvements by IFNAR1 blocking antibody (anifrolumab) in patients with type I interferonopathies. J Clin Immunol. (2025) 45(1):31. 10.1007/s10875-024-01826-2 PMC11499543 39441221

[8] Fushida N Horii M Oishi K Matsushita T. Anifrolumab for systemic lupus erythematosus: a clinical study of Japanese patients in Kanazawa university hospital. J Dermatol. (2024) 51:607–11. 10.1111/1346-8138.17027 37929294

[9] Shmizu M Kaneko S Shimbo A Hatano M Miyaoka F Irabu H. Anifrolumab for refractory cutaneous lupus lesions in pediatric systemic lupus erythematosus. Lupus. (2025) 34:533–6. 10.1177/09612033251330094 40123095

